# Removal of Dr. Newman from Buffalo

**Published:** 1860-01

**Authors:** 


*Removal of Dr. Newman from Buffalo.*—In the Proceedings of the Buffalo Medical Association, which appear in this number, our readers will see that Dr. James M. Newman, whose name is familiar to them as one of the most able contributors the Journal has ever had, has removed from the city of Buffalo. The removal of Dr. Newman will be much felt by the profession and citizens of Buffalo, who are greatly indebted to his industry and talents, for the prosperity and standing of many of the medical institutions of their city, in which they are almost equally interested. To those with whom he has been thrown in daily contact, we can only say that we appreciate the loss which they have suffered; no one has more industriously and unselfishly labored for the general good than he. And to the profession of Lowell, with whom the doctor has now cast his lot, we wish to offer our congratulations, knowing that he cannot fail to become as much loved and respected by them as he has been in his old home.

Dr. Newman has always taken a deep interest in the medical societies of the city and county, and has been emphatically one of their working men. Not only has he done much toward the scientific character of their meetings, but in their business affairs he has been most active and efficient. He was, for a long time, the Secretary of both the Erie County Society and the Buffalo Medical Association—a position which entailed upon him no little labor, especially as he instituted an order in the records and affairs of these bodies, such as few could or would have effected. He was one of the most efficient men in the re-organization of the Buffalo Medical Association, and in the organization of the Physicians' Charitable Fund Association, the establishment of which we noticed some months ago. Dr. Newman has also been active in the State Medical Society, and in the American Medical Association;—having presented to the latter body in 1856 an elaborate report on the "Sanitary Police of Cities;" a paper which added considerably to his reputation. This report is to be found in Vol. IX of the Proceedings of the Association, and has been highly appreciated by the profession. While speaking of this, we cannot forbear to mention the practical benefit to the city of Buffalo from the labors of Dr. Newman in this department. As city physician, he did much in improving the sanitary condition of the city, doing an amount of effective labor which, as intimated by one of the daily papers of Buffalo, contrasts very strongly with the use that some have made of that important office.

We had the pleasure of being associated with Dr. Newman, as member of the Medical Board of the Buffalo General Hospital, and can bear testimony to the services which he has rendered to that institution. The hospital is now in active operation, with one wing, constituting one-third of its elegant building, already completed; and we venture to say, that its present position is in a great measure due to the indefatigable exertions of Dr. Newman. Those only who have been personally engaged in such an enterprise, can appreciate the difficulties to be overcome in establishing a medical institution of any kind. The bitter hostility which it must always experience, and the apathy of those not unfavorably disposed towards it, make the undertaking disheartening, as well as difficult. Through all these trials has Dr. Newman labored for the hospital; and we are confident that he must feel the deepest regret at severing his connection with it, when beginning to see the fruits of his exertions.

As a journalist, we have always received the most valuable assistance from the pen of Dr. Newman, and we hope and expect that his change of residence will not make him a less frequent contributor to our pages. We heartily wish him the success in his new sphere of action, of which, by his talent and acquirements, he is so worthy.

